# Complete genome sequence of the *Exiguobacterium* bacteriophage

**DOI:** 10.1128/mra.00342-24

**Published:** 2024-06-11

**Authors:** Erika Camacho-Beltrán, Juan José Morales-Aguilar, Melina López-Meyer, Gabriel Rincón-Enríquez, Evangelina Esmeralda Quiñones-Aguilar

**Affiliations:** 1 Laboratorio de Fitopatología, Unidad de Biotecnología Vegetal, Centro de Investigación y Asistencia en Tecnología y Diseño del Estado de Jalisco, A.C. Zapopan, Jalisco, Mexico; 2 Instituto Politécnico Nacional, Centro Interdisciplinario de Investigación para el Desarrollo Integral Regional (CIIDIR-Unidad Sinaloa), Guasave, Sinaloa, Mexico; 3 Universidad Autónoma de Occidente, Unidad Regional Guasave. Avenida Universidad S/N Colonia Villa Universidad, Guasave, Sinaloa, Mexico; Portland State University, Portland, Oregon, USA

**Keywords:** *Exiguobacterium* acetilycum, virus, biocontrol, disease of bean crops

## Abstract

We purified a lytic bacteriophage from soil collected in Guasave, Sinaloa: phiExGM16. This bacteriophage was isolated using the host, *Exiguobacterium acetilycum*. Its 17.6 kb genome contains 33 putative genes and shows a cover of 64% with 76.37% of nucleotide identity to *Microbacterium phage* Noelani.

## ANNOUNCEMENT

**Fig 1:**
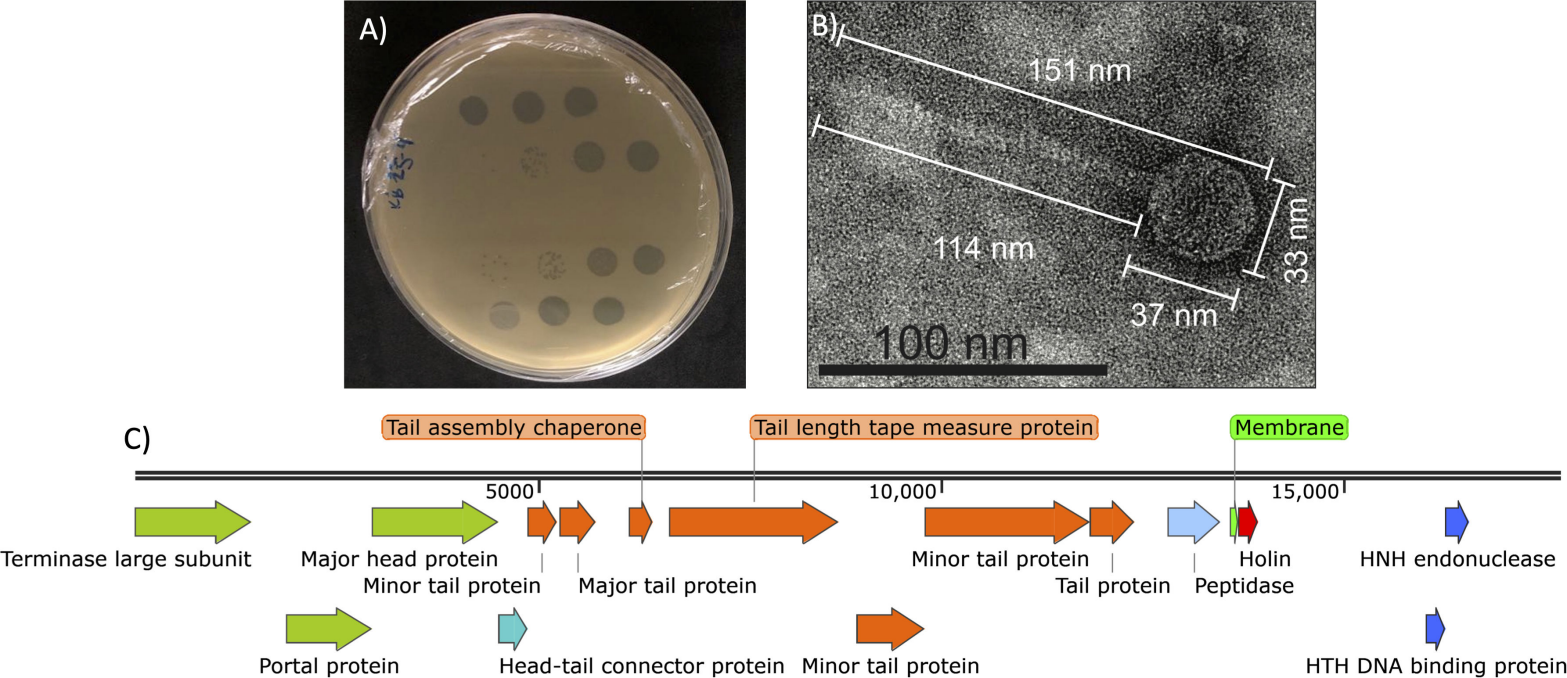
Characterization of the *Exiguobacterium* bacteriophage phiExGM16. (**A**) phiExGM16 forms small clear plaques. (**B**) An electron microscopic image highlights the *Siphoviridae* morphology of phiExGM16, with an icosahedral capsid (diameter, ~33 nm) attached to a noncontractile tail (length, ~114 nm). The sample was viewed at an accelerating voltage of 200 kV with a LaB6 transmission electron microscope (JEOL, JEM 2100) after it was fixed with 1% glutaraldehyde on a freshly glow-discharged Formvar/carbon-coated copper grid for 10 min and stained with 1% aqueous uranyl acetate for 1 min. (**C**) The phiExGM16 genome shows 16 annotated putative protein-coding genes with known functions. Colors are assigned to expected functions according to annotation information. Green (head and packing), turquoise (connector), orange (tail), fluorescent green (membrane), red (lysis), blue (nucleotide metabolism), and light blue (other functions). The image was created with SnapGene software.

Bacteriophages are the most abundant organisms on Earth (1). The discovery and genome analyses of bacteriophages facilitate their wide-ranging uses. Lytic phages are essential for new developments of biotechnological tools as biological control agents. Studies of the genes and biology of lytic phages are essential for the new agricultural organic era. Bacteriophage phiExGM16 was isolated from soil samples collected on commercial fields of common bean var. Azufrado Higuera, with symptoms associated with bacterial wilt disease (Guasave, Sinaloa, México, 25.544444 N, 108.376389 W) on 12 January 2022. The sample was taken 0 to 20 cm below the surface of the soil. The soil sample (100 g) was suspended in King agar B medium (100 mL) (2), inoculated with *Exiguobacterium acetilycum* (600 µL overnight-culture; laboratory strain BV25 identified with 16S rDNA sequencing), and incubated for 24 h at 28°C. The resulting slurry was centrifuged (8,000 *× g*, 20 min), and the supernatant was filtered (0.22 mm). Single plaques were isolated and purified two times (3). phiExGM16 forms small clear plaques (Fig. 1A). The phage morphology was visualized using negative staining with uranyl acetate (4). phiExGM16 exhibits a siphovirus morphology (Fig. 1B). BV25 cells infected at an MOI of 1 and incubated for 18 h were lysate, spun, and filtered before DNA extraction. The genomic DNA of a phage lysate was extracted using the phenol-chloroform method (5). The phage genome was digested with DNase I (04356282001, Roche) and S1 nuclease (N5761, Promega), but not with RNase A (R6148, Sigma-Aldrich) or exonuclease III (M1811, Promega) used according to manufacturer’s instructions. For the complete genome analysis of phiExGM16, a DNA genomic library was prepared with the MiSeq Reagent Nano Kit v2 (MS-103-1001, Illumina). High-throughput sequencing was performed with a Miseq instrument (SY-410-1003, MiSeq; Illumina) with a 2 × 150 bp maximum read length, which resulted in 3,061,301 paired-end 150 bp raw reads. The genome was assembled using the Illumina A5-miseq pipeline (6), resulting in a contig of 17,662 bp with a G+C content of 68%, and average coverage of 219 was obtained, then the accuracy of the genome was checked using QUAST (7), showing no break point in the contig and PhageScope (8), showing 100% of completeness and high-quality. The genome was manually reviewed and then annotated using pharokka embedded in Galaxy Server (9) and PhageScope (8), identifying 33 putative genes and no tRNA genes. Putative functions could be assigned to 16 genes, including head and tail assembly genes, holin gen (10), RNA, and nucleotide metabolism (Fig. 1C). A proteins BLAST analysis (Database nr) (11) was performed in some of the proteins in phiExGM16 bacteriophage such as the major head protein, holin, and terminase large subunit presenting 75%, 81%, and 84% of amino acid identity to *Microbacterium* phage proteins accession numbers QKY80443.1, YP_009996604.1, and QKY79133.1, respectively, also the major tail protein shows 74% of amino acid identity to *Microbacterium* phage protein accession number YP_009996744.1, and the portal protein showing 83% of amino acid identity to *Microbacterium* phage protein accession number URM86188.1. BLAST analysis (Database nr/nt) (12) of the complete genome generated a best hit with a cover of 64% with 76.37% of nucleotide identity to *Microbacterium* phage Noelani NC_052937.

## Data Availability

The genome sequence of bacteriophage phiExGM16 was deposited under GenBank accession number PP475795. The raw sequence reads are available in the SRA database with the accession number SRP492678 (BioProject accession number PRJNA1081932).

## AFTER EPUB

[This article was published on 11 June 2024 with incorrect spelling of *Exiguobacterium*. The spelling was corrected in the current version, posted on 26 June 2024.]
